# Using Unmanned Aerial Vehicles in Postfire Vegetation Survey Campaigns through Large and Heterogeneous Areas: Opportunities and Challenges

**DOI:** 10.3390/s18020586

**Published:** 2018-02-14

**Authors:** José Manuel Fernández-Guisuraga, Enoc Sanz-Ablanedo, Susana Suárez-Seoane, Leonor Calvo

**Affiliations:** 1 Biodiversity and Environmental Management Department, Faculty of Biological and Environmental Sciences, University of León, 24071 León, Spain; s.seoane@unileon.es (S.S.-S.); leonor.calvo@unileon.es (L.C.); 2 Mining Technology, Topography and Structures Department, University of León, 22400 Ponferrada, Spain; enocsanz@unileon.es

**Keywords:** drone, megafire, multispectral imagery, Parrot SEQUOIA, UAV, WorldView-2

## Abstract

This study evaluated the opportunities and challenges of using drones to obtain multispectral orthomosaics at ultra-high resolution that could be useful for monitoring large and heterogeneous burned areas. We conducted a survey using an octocopter equipped with a Parrot SEQUOIA multispectral camera in a 3000 ha framework located within the perimeter of a megafire in Spain. We assessed the quality of both the camera raw imagery and the multispectral orthomosaic obtained, as well as the required processing capability. Additionally, we compared the spatial information provided by the drone orthomosaic at ultra-high spatial resolution with another image provided by the WorldView-2 satellite at high spatial resolution. The drone raw imagery presented some anomalies, such as horizontal banding noise and non-homogeneous radiometry. Camera locations showed a lack of synchrony of the single frequency GPS receiver. The georeferencing process based on ground control points achieved an error lower than 30 cm in X-Y and lower than 55 cm in Z. The drone orthomosaic provided more information in terms of spatial variability in heterogeneous burned areas in comparison with the WorldView-2 satellite imagery. The drone orthomosaic could constitute a viable alternative for the evaluation of post-fire vegetation regeneration in large and heterogeneous burned areas.

## 1. Introduction

Natural hazards, such as wildfires, constitute a serious global concern that is expected to increase in the future [1] mainly due to global warming predictions and changes in land use [2,3]. In particular, the increasing severity and recurrence of large forest fires in Mediterranean Basin ecosystems [4] can lead to severe long-term land degradation, including desertification [5,6]. Thus, post-fire monitoring of these systems through different tools should be a priority for management purposes [7].

Advances in geospatial technologies have led to an increase in the utilization of remote sensing techniques [3], which represent a major opportunity for conducting post-fire surveys in large and heterogeneous burned ecosystems [8]. High spatial resolution satellite imagery, such as that provided by Deimos-2, GeoEye-2, QuickBird or WorldView-2 on-board sensors, among others, have been used to assess post-fire regeneration in terms of fractional vegetation cover [8], species richness [9] or the basal area of tree species [10]. Nevertheless, satellite imagery shows certain weaknesses that could limit its applicability in the post-fire monitoring of highly heterogeneous and dynamic areas. First, the revisit periods of satellite platforms cannot be user-controlled for short-term time series monitoring of plant communities with a fast regeneration rate, such as shrublands, after the occurrence of a disturbance [11]. Second, satellite imagery may be seriously affected by cloud cover and its projected shadows [12]. Third, even though the spatial resolution of multispectral satellite imagery has been improved, resolutions below one meter have not been achieved, which could become a problem when monitoring certain biophysical properties in spatially heterogeneous ecosystems [11]. For their part, sensors aboard manned platforms such as aircrafts, can also be used to conduct post-fire surveys on demand, but regular monitoring is constrained because of the high economic costs [13]. 

The use of lightweight unmanned aerial vehicles (UAVs) usually implies lower economic costs than other remote sensing techniques when surveying relatively small areas [11,13,14,15,16] and their low flight speed and flight altitude enables ultra-high spatial resolution (better than 20 cm) imagery [11] to be taken. In burned areas, vegetation recovery is not homogeneous due to the spatial variation in fire regime, pre-fire plant community composition and environmental characteristics [17]. Therefore, the use of ultra-high spatial resolution imagery would allow for conducting studies at the population level [18] to assess the effectiveness of post-fire management actions such as seedlings plantation strategies or seedling recruitment. Moreover, UAVs are flexible in terms of attaching different kinds of sensors (e.g., RGB, multispectral or LiDAR), also allowing the operator to schedule the exact flight time to gather data over target areas [19]. Nevertheless, UAV imagery may be difficult to manage because of its ultra-high spatial resolution [20] and the platform does not allow for a simultaneous image acquisition of large areas [19] and has a limited flight time [12].

Several research projects have used UAV on-board sensors for wildlife population monitoring [21,22,23,24,25,26], estimation of forest structural parameters [27,28,29,30,31,32], individual tree or species mapping [33,34,35,36], estimation of post-fire vegetation recovery from digital surface models [37], estimation of fire severity [18,38] and forest fire detection [39]. However, to our knowledge, the operational and processing challenges in the generation of multispectral mosaics derived from rotor-based UAVs imagery that allow very large burned areas to be monitored have not been assessed yet. In addition, it would be necessary to know the pros and cons of this tool on large burned surfaces in relation to fine-grained satellite imagery. In this context, the comparison between the spatial and spectral information provided by UAVs and satellites on heterogeneous surfaces would be essential to determine their suitability for representing fine scale ground variability. Some authors have compared the spatial information provided by ultra-high spatial resolution imagery captured by UAVs and high spatial resolution satellite imagery, for instance that provided by WorldView-2 satellite on agricultural systems such as vineyards [12] or crops [40], but not to our knowledge in heterogeneous burned areas.

Most common cameras employed in UAV surveys are digital RGB cameras [11,40] or digital cameras where one of the visible bands has been adapted for NIR imagery acquisition [16,32]. Also, multispectral cameras, such as Tetracam ADC Lite [41] or MicaSense RedEdge [42], have been chosen to perform aerial surveys. For its part, the Parrot SEQUOIA (Parrot SA, Paris, France) is a novel and affordable multispectral camera released on the market in early 2016, whose imagery quality has not been evaluated in scientific literature.

The main objective of this study is to evaluate the feasibility of using a rotor-based UAV with an on-board multispectral sensor (Parrot SEQUOIA) to obtain a multispectral orthomosaic at ultra-high spatial resolution, which could be useful for forestry management purposes in a heterogeneous and large burned area (3000 ha). Specifically, we intend to: (1) evaluate the quality of the raw imagery dataset captured with the Parrot SEQUOIA multispectral camera; (2) discuss the challenges encountered when dealing with the volume of data at ultra-high spatial resolution generated in the UAV survey carried out in a large area, and assess both the required processing capability and the quality of the obtained multispectral mosaic; and (3) compare the spatial information provided by the UAV ultra-high resolution multispectral mosaic with high spatial resolution satellite imagery (WorldView-2) in a heterogeneous burned landscape.

## 2. Materials and Methods

### 2.1. Study Area

**Figure 1:**
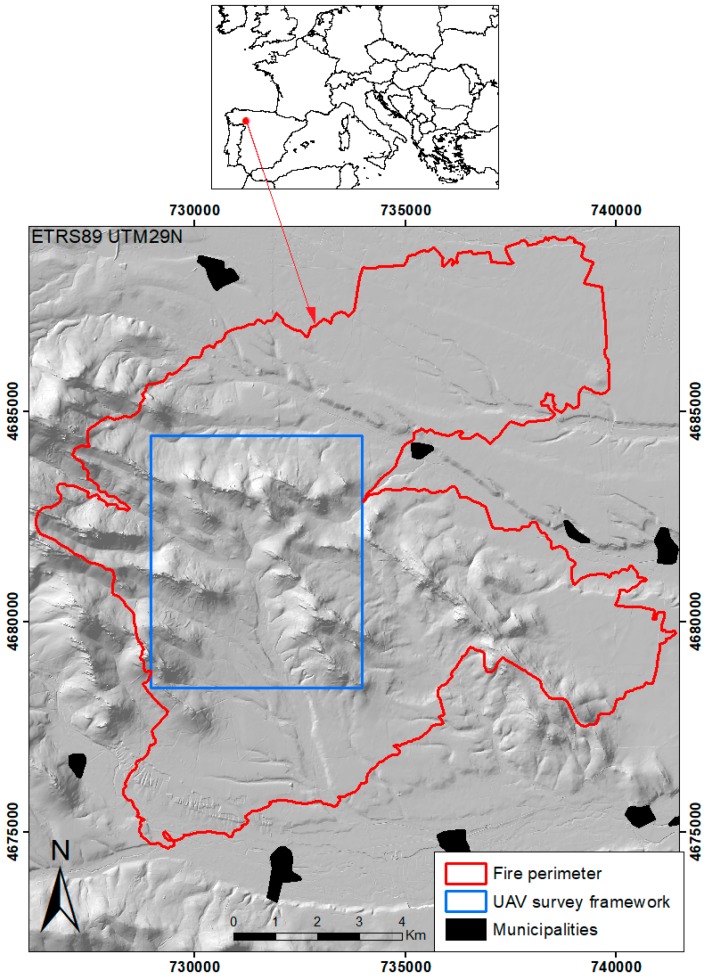
UAV survey framework within the megafire perimeter of Sierra del Teleno.

The study area (Figure 1) is a 3000 ha framework located in the central section of a megafire of about 10,000 ha which occurred in a *Pinus pinaster* stand in Sierra del Teleno (León Province, northwest Spain) in August 2012. The survey framework was representative of the heterogeneity of the fire regime within the perimeter.

The study area is dominated by an Appalachian relief with prominent quartzite crests, wide valleys with moderate slopes on the upper two thirds of the study area, and sedimentary terraces on the lower third. The mean annual temperature in the area is 10 °C, with an average rainfall of 650 mm. The understory plant community after the occurrence of the megafire is composed by species such as *Halimium alyssoides*, *Pterospartum tridentatum* and *Erica australis* [43], with a great regeneration of *Pinus pinaster* seedlings.

### 2.2. UAV Platform and Multispectral Camera

**Figure 2:**
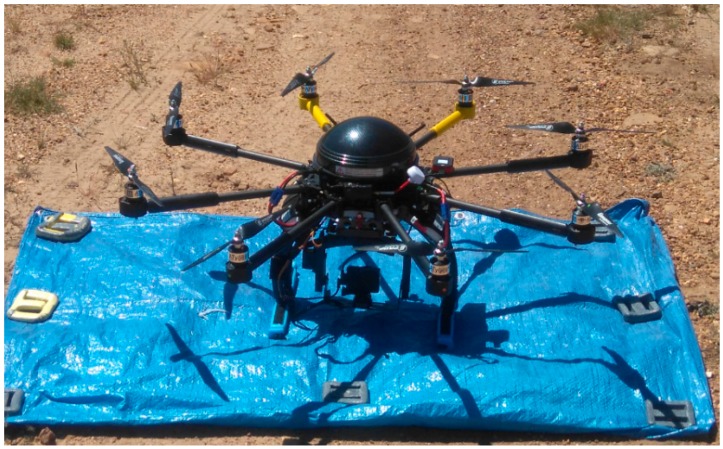
ATyges FV8 octocopter used for the aerial survey.

A FV8 octocopter (ATyges, Málaga, Spain, Figure 2) was chosen to perform the aerial survey of a large burned surface of 3000 ha. This UAV is manufactured entirely from carbon fiber and titanium and it weighs 3.5 kg, with a maximum payload mass of 1.5 kg. The eight brushless motors (AXI-ATYGES 2814/22 260 W with a maximum efficiency of 85%) are powered by two lithium-ion polymer batteries (rated capacity and voltage of 8200 mAh and 14.8 V, respectively). The UAV has a cruising speed of 7 m·s^−1^ (10 m·s^−1^ max), with an ascent/descent rate of 5.4 km·h^−1^ (10.8 km·h^−1^ max). The maximum interference-free flight range is 3 km, with a flight duration of 10–25 min depending on the payload and weather conditions. The maximum flight height is 500 m above ground layer (AGL). The platform is remotely controlled by a 12-channel MZ-24 HoTT radio transmitter (Graupner, Kirchheim unter Teck, Germany) operating at 2.4 GHz. The UAV is equipped with a micro FPV camera with real-time video transmission at 5.8 GHz to a Flysight monitor. The core component of the UAV electronics is an ATmega 1284P flight controller (Microchip Technology Inc., Chandler, AZ, USA) with an integrated pressure sensor, gyroscopes and accelerometers. The navigation control board is based on an Atmel ARM9 microcontroller and it has a MicroSD card socket for waypoint data storage. The GPS module with integrated antenna is a LEA-6 (u-blox, Thalwil, Switzerland). This system allows for autonomous, semi-autonomous and manual takeoffs, landings and flight.

A Parrot SEQUOIA multispectral camera was installed underneath the UAV platform. The camera has four 1.2-megapixel monochrome sensors that collect global shutter imagery along four discrete spectral bands [44]: green (center wavelength -CW-: 550 nm; bandwidth -BW-: 40 nm), red (CW: 660 nm; BW: 40 nm), red edge (CW: 735 nm; BW: 10 nm) and near infrared -NIR- (CW: 790 nm; BW: 40 nm). The horizontal (HFOV), vertical (VFOV) and diagonal (DFOV) fields of view of the multispectral camera are 70.6°, 52.6° and 89.6°, respectively, with a focal length of 4 mm. With a flight altitude of 120 m, a ground sample distance (GSD) of 15 cm can be achieved. The camera was bundled with an irradiance sensor to record light conditions in the same spectral bands as the multispectral sensor. The weight of the multispectral camera plus the irradiance sensor is 107 g. 16-bit RAW files (based on 10-bit data) are stored in this camera during image shooting. ISO value and exposure time was set to automatic. Every image capture setting is saved in a text metadata file together with the irradiance sensor data. All this information is taken into account during the preprocessing stage to obtain absolute reflectance values for the final product.

### 2.3. UAV Survey Campaign

The aerial survey campaign was conducted for 100 h between June and July 2016. All flights (383) were performed within a 6-h window around the solar zenith to maintain relatively constant lighting conditions. Though small variations in environmental conditions were rectified with the irradiance sensor, severe wind or cloud cover were avoided.

Mikrokopter Tools software was used to plan flights, which allowed the operator to generate an automatic flight route with waypoints depending on the camera’s field of view (FOV), the chosen forward and side overlap between images and the required GSD [45]. A digital elevation model (DEM) was used to keep the same distance AGL in all flights tracks owing to the large difference in altitude (410 m) in the study framework. Flight tracks were uploaded in the UAV for each complete day. The flight height was fixed at 120 m AGL, providing an average ground resolution of 14.8 cm·pixel^−1^ given the specific camera characteristics. Each flight had an effective duration of 5–6 min (without including the takeoff and landing), with an average speed of 10 m s^−1^. Battery change time and time needed to reach each takeoff site were not computed. However, both time lapses were included in the total flight time of 100 h. The camera trigger interval was set to a platform advance distance of 22.4 m in order to achieve an 80% forward image overlap at the fixed flight altitude. The waypoints route planned allowed an 80% side image overlap. The image overlap between adjacent flights was at least a flight line. The quality of the raw imagery dataset acquired during the UAV survey was evaluated to search for potentially undesired anomalies, such as: (1) horizontal banding noise (HBN) [46]; (2) non-homogeneous radiometry and issues related with hot-spot or opposition effect [47] or (3) blurring effects [48].

### 2.4. Image Data Processing

UAV imagery was processed into a multispectral mosaic with Pix4Dmapper Pro 3.0 [49] following the “Ag Multispectral” template. This software integrates computer vision techniques with photogrammetry algorithms [50] to obtain high accuracy in aerial imagery processing [51,52]. Pix4Dmapper Pro computes keypoints on the images and uses them to find matches between images. From these initial matches, the software runs several automatic aerial triangulation (AAT), bundle block adjustments (BBA) and camera self-calibration steps iteratively until optimal reconstruction is achieved. Then, a densified point cloud is generated to obtain a highly detailed digital surface model (DSM) that will be used to generate the reflectance maps. A pre-process or normalization was automatically applied to the imagery, where 16 bits TIF files (10 bit RAW data) were converted to standard 8 bit jpg files, taking into account the ISO, exposure time and irradiance sensor data.

**Figure 3:**
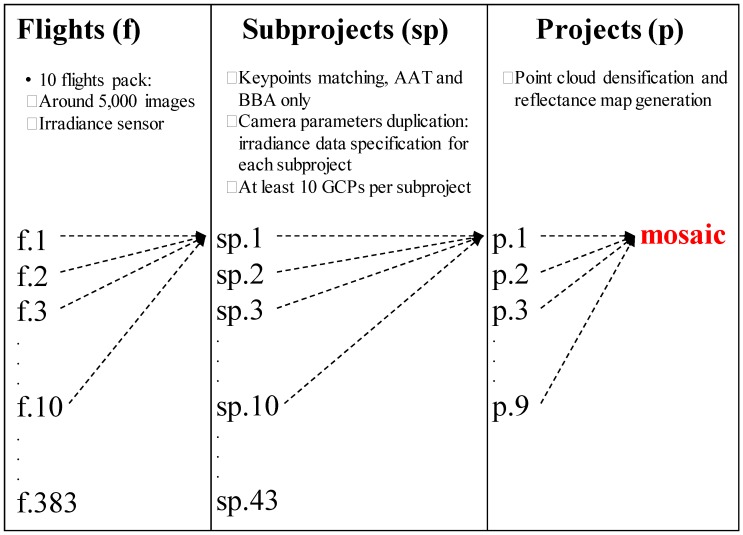
Processing workflow of the UAV imagery with Pix4Dmapper Pro.

A high-end computer with a 12-core Intel i7 processor and 64 GB of RAM was used to process the imagery. Most of the processing steps in Pix4Dmapper Pro need a large number of computational resources that grow exponentially as more images are simultaneously processed. Due to software and hardware limitations for very large projects (above 10,000 images), each of the nine projects was split into smaller subprojects. The subprojects could then be merged after completing the AAT-BBA stage for each one, being necessary only to further process the less demanding subsequent steps for the merged project. Flights, subprojects and projects processing workflows are detailed in Figure 3. Radiometric corrections were introduced based on camera setup parameters and sun irradiance measured by the irradiance sensor. Initial georeferencing was achieved by introducing camera locations in the AAT-BBA stage. At least ten ground control points (GCPs) evenly distributed per subproject were extracted from aerial orthophotos of the Spain National Plan of Aerial Orthophotography (PNOA) to improve global spatial accuracy. This dataset has a GSD of 25 cm with an accuracy better than 0.50 m in terms of RMSE_X,Y_ [53]. The multispectral outputs (four reflectance maps with a GSD of 20 cm) of the Pix4D projects were mosaicked using ArcGIS 10.3.1 (Esri, Redlands, CA, USA) [54] without applying reflectance normalization to avoid the modification of the computed reflectance values in the radiometric correction process. Geospatial accuracy of the outputs was assessed in terms of root mean square error (RMSE) in X, Y and Z from the coordinates of 50 targets uniformly arranged through the UAV survey framework. The X, Y and Z coordinates of the Control Points (CPs) were measured with a high-accuracy GPS receiver (Spectra Precision MobileMapper 20 with accuracy better than 0.50 m in terms of RMSE_X,Y_) in postprocessing mode.

### 2.5. WorldView-2 High Spatial Resolution Satellite Imagery and Image Comparison Statistical Analysis

A WorldView-2 image acquired on 23 June 2016 for the study framework was used to compare the spatial information provided by the UAV platform with high resolution satellite imagery in a heterogeneous burned landscape. The spatial resolution of the multispectral sensor on-board WorldView-2 satellite at nadir is 1.84 m, but the image was delivered by DigitalGlobe resampled to 2 m. This sensor has eight bands in the visible and NIR region of the spectrum [55]: coastal blue (400–450 nm), blue (450–510 nm), green (510–580 nm), yellow (585–625 nm), red (630–690 nm), red edge (705–745 nm), NIR1 (770–895 nm) and NIR2 (860–1040 nm). The raw image was orthorectified with a DEM (accuracy better than 20 cm in terms of RMSE_Z_) and GCPs extracted from PNOA orthophotos. The image atmospheric correction was conducted by the Fast Line-of-sight Atmospheric Analysis of Spectral Hypercubes (FLAASH) algorithm [56]. The HyPARE algorithm implemented in ENVI 5.3 [57] was used to geometrically align the UAV multispectral orthomosaic and the WorldView-2 image achieving a UAV subpixel RMSE (<20 cm).

The image comparison was performed on the basis of the reflectance values and the Normalized Difference Vegetation Index (NDVI) of the UAV multispectral orthomosaic and the WorldView-2 image. UAV multispectral mosaic at original resolution (20 cm) was resampled to a GSD of 1 m (half of WorldView-2 spatial resolution) and 2 m (WorldView-2 spatial resolution) with a block average function for the input pixels within a set of non-overlapping windows with the required size (5 × 5 and 10 × 10 pixels). The function was computed with ArcGIS 10.3.1. Pearson bivariate correlations between the UAV multispectral mosaic (GSD of 20 cm, 1 m and 2 m) and WorldView-2 image (GSD of 2 m) were calculated on each comparable band to assess the spatial information provided by each sensor in our survey framework. To determine the reflectance variability between sensors, we computed the variance in the reflectance values in each band of the UAV images (native spatial resolution and resampled) and WorldView-2 image. For the more heterogeneous surface within the survey framework, which covers 1.5 ha, a basic statistic package was calculated on the UAV (at native resolution and 2 m) and WorldView-2 NDVI maps to compare the potentiality of these products in post-fire vegetation monitoring.

## 3. Results

### 3.1. Raw Imagery Dataset Quality

**Figure 4:**
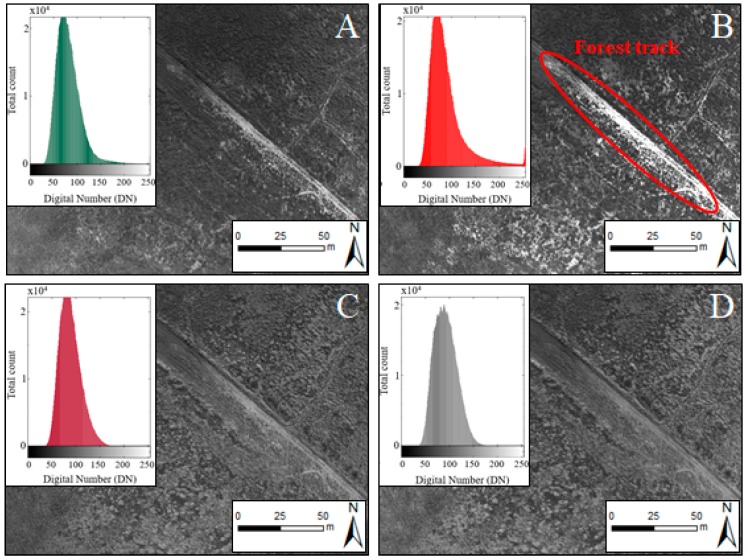
Example normalized UAV images from the dataset corresponding to green (**A**); red (**B**); red edge (**C**) and NIR (**D**) bands, as well as the image histograms.

From 383 UAV flights, we acquired 45,875 images for each band, which made a total of 183,500 raw images that represented approximately 430 GB of information. The normalized UAV images had a balanced contrast. However, the red channel showed some saturation over highly reflective surfaces on this wavelength, such as forest tracks in our study area (Figure 4).

**Figure 5:**
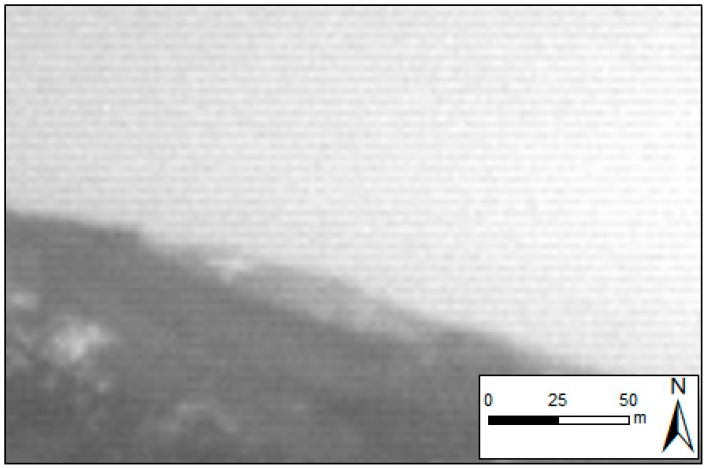
Horizontal banding noise in a raw green channel image.

A slightly horizontal banding noise (HBN) was observed within the four channels of the camera, especially in the green channel (Figure 5). The banding effect was more noticeable at the top and bottom of the image, where differences in the digital levels of alternate rows representing the same object were higher than 10%.

**Figure 6:**
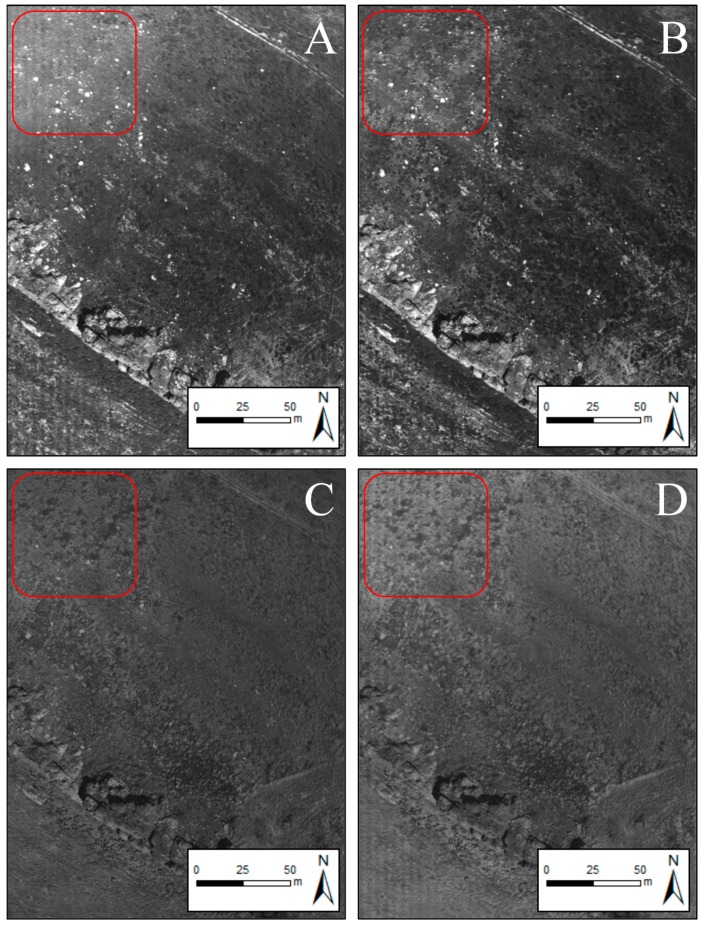
Hot-spot effect on green (**A**); red (**B**); red edge (**C**) and NIR (**D**) bands. Note that the upper left corner (framed in red) has higher reflectance values regardless of the terrain characteristics.

Another undesired effect observed across the imagery was non-homogeneous radiometry across the image related with Bidirectional Reflectance Distribution Function (BRDF) [47]. In particular, a specific area of the imagery had systematically higher reflectance values than the remaining areas (Figure 6). This radiometric anomaly effect is commonly denominated hot-spot or opposition effect [58,59] and it appears as a consequence of the camera and sun position alignment [60]. For its part, the image dataset did not exhibit blurring effects that are usually associated with camera shaking [15].

### 3.2. Multispectral Mosaic Processing and Product Quality

The processing of the multispectral orthomosaic was labor-intensive and time-consuming because of the large size of the surveyed area [19] and the ultra-high spatial resolution of the dataset [11]. Each subproject took 3–6 h to process the AAT, BBA and camera self-calibration. Point cloud densification and generation of the reflectance maps took up to 14 h for each project. The total amount of time required to process the whole dataset was about 320 h (20 days) with the available processing resources, including software failures.

**Figure 7:**
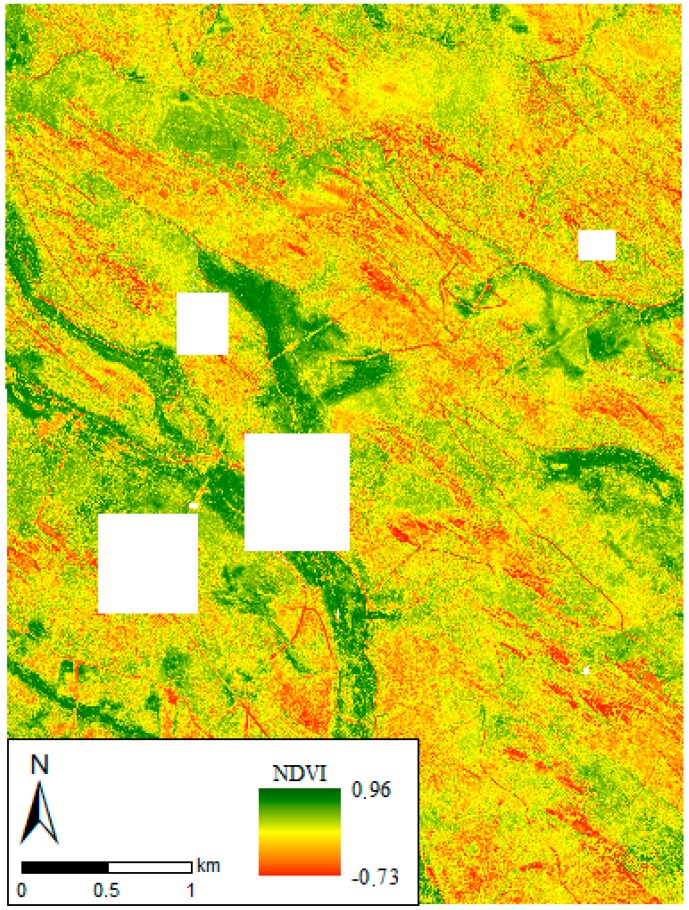
NDVI mosaic of the UAV survey framework. Blank areas are those masked due to the malfunction of the irradiance sensor.

For each of the 43 subprojects, the 3D reconstruction algorithm (AAT, BBA and self-calibration) obtained between 95% and 99% images aligned on the basis of more than 10,000 keypoints extracted from each image, with over 5500 keypoints matching with at least another two adjacent images. Green and NIR channels obtained the highest number of matches, whereas red channel systematically got the lowest number. The total number of 2D keypoint observations for BBA in each subproject was about 9 million, whereas the number of 3D matching points was 1.5 million, with a mean reprojection error of 0.2–0.3 pixels. The large forward and side overlap provided high accuracy in the keypoint matching step between adjacent images, as [45] pointed out. Changes between nominal and final parameters defining the geometrical model of the camera were as low as 0.01%. For its part, the point cloud densification at the merge step of the subprojects obtained between 6 × 10^6^ and 7 × 10^6^ 3D densified points. For each of the nine projects, four reflectance maps (green, red, red edge and NIR) were obtained with a resampled GSD of 20 cm/pixel. Some areas of these maps were excluded (Figure 7) due to reflectance anomalies caused by USB-disconnections between the camera and the irradiance sensor.

Initial georeferencing was achieved by introducing the UAV’s GPS positions taken at each camera shot in the bundle-block adjustment process within Pix4D workflow. The precision reported by Pix4D, calculated as the root mean square error (RMSE), was between 1.5–3 m in X-Y and between 2–4 m in Z.

The final georeferencing of the subprojects achieved by using ground control points (GCPs) extracted from PNOA orthophotos achieved an RMSE lower than 30 cm in X-Y and lower than 55 cm in Z. Horizontal and vertical accuracy was improved from initial georeferencing at least 80% and 73% respectively, after providing evenly distributed GCPs through the UAV survey framework.

### 3.3. Comparison of the Spatial Information Provided by UAV and WorldView-2 Imagery

Higher r_Pearson_ values were obtained when the UAV mosaic resolution approached the resolution of the WorldView-2 image (2 m) (Table 1) for each band of the spectrum. The correlation between the two remote sensing platforms for each resolution was stronger for the visible region of the spectrum.

The largest variance in the reflectance values of each band was found for the UAV orthomosaic at 20 cm spatial resolution (Table 2). The variance of the UAV orthomosaic at 2 m of spatial resolution was similar to that of the WorldView-2 image.

**Figure 8:**
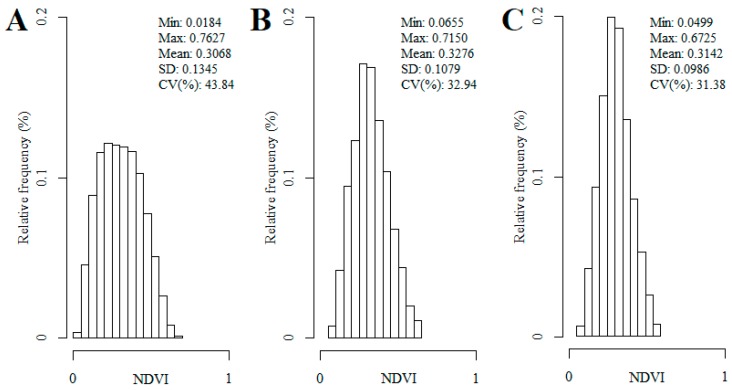
Histogram and statistics of the UAV (**A**) and WorldView-2 (**B**) NDVI map at native resolutions, and of the UAV NDVI map resampled to 2 m (**C**), corresponding to a 1.5 ha portion within pine plantation area.

**Figure 9:**
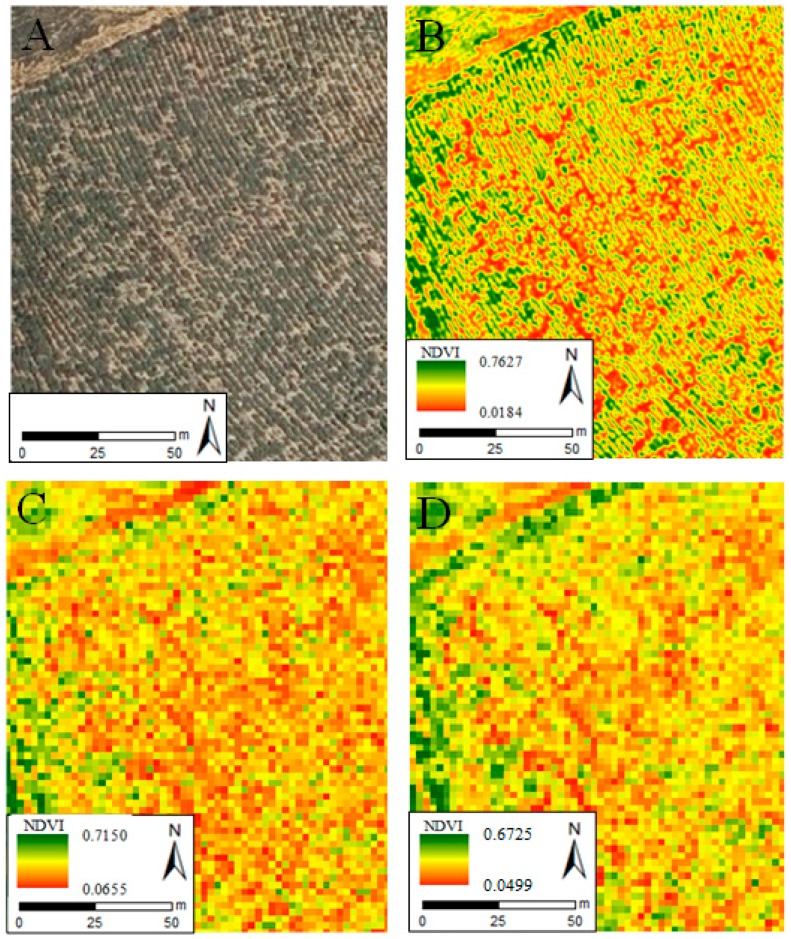
PNOA orthophoto (**A**), UAV (**B**) and WorldView-2 (**C**) NDVI maps at original resolutions, and UAV NDVI map resampled to 2 m (**D**), corresponding to a heterogeneous surface of 1.5 ha within the survey framework.

The comparison between UAV and WorldView-2 NDVI maps derived from the imagery datasets at the original resolution of each sensor, corresponding to a heterogeneous surface of 1.5 ha within the survey framework, revealed greater variability in the UAV pixel values (Figure 8A,B). The horizontal structure of the vegetation observed in this area (Figure 9A) can be identified in the UAV mosaic (Figure 9B), but not in the WorldView-2 image (Figure 9C). The UAV NDVI map resampled to 2 m presented similar variability to the WorldView-2 image (Figure 8B,C).

## 4. Discussion

This study evaluated the strengths and limitations of using a rotor-based UAV equipped with a novel multispectral camera (Parrot SEQUOIA) to conduct a field survey of a large (3000 ha) and heterogeneous burned surface. Our results indicate that the ultra-high spatial resolution UAV multispectral orthomosaic represents a valuable tool for post-fire management applications at fine spatial scales [18]. However, due to the ultra-high spatial resolution of the data and the large size of the surveyed area, data processing was highly time consuming.

Multispectral cameras onboard UAVs provide countless opportunities for remote sensing applications, but the technological limitations of these sensors [46] would require evaluation of the quality of the captured raw imagery data, particularly in novel sensors. In this study, we found that the raw imagery captured by the Parrot SEQUOIA multispectral camera presented some undesired radiometric anomalies. In the red channel we observed sensor saturation over highly reflective surfaces. This effect was not induced by radiometric down sampling from 10 to 8-bit performed by Pix4D during processing because it was present both in raw (10-bit) and in normalized (8-bit) images. The horizontal banding noise observed within the four channels of the camera is a common artifact of CMOS (complementary metal oxide semiconductor) rolling shutter sensors [46]. However, the Parrot SEQUOIA uses a global shutter system and this effect should not be significant in this multispectral sensor. To our knowledge, this camera has not been used in previous scientific studies and, therefore, this issue has not been reported so far. The issues related with Bidirectional Reflectance Distribution Function (BRDF) effect are magnified in sensors with a wide Field of View [61,62] such as the Parrot SEQUOIA. For its part, the hot-spot or opposition effect was more apparent at shorter wavelengths, as also highlighted by [47]. Some corrections to mask this effect have been proposed [59] that must be made individually for each image taking into account the time and position of the image acquisition, image orientation and solar positioning (azimuth and elevation), following some photogrammetric steps. Thus, the correction of this radiometric anomaly as well as the BRDF effect is very challenging and time consuming, becoming an unapproachable task when dealing with large imagery datasets [58]. The absence of a blurring effect in our dataset could be explained by the increased flight stability that the rotor-based UAVs offer over fixed-wing UAV platforms, also exhibiting fewer vibrations [13,29]. Moreover, the Parrot SEQUOIA camera was attached to the platform with a rubber damper to minimize vibrations, and the camera acquired imagery with the focal length set to infinity and fast shutter speed [15], preventing the occurrence of this effect. USB-disconnections between the camera and the irradiance sensor could be associated to a poor connection. However, the disconnections did not imply a major problem with the irradiance sensor, considering that it provided complete records for more than 90% of the survey framework with varying atmospheric conditions between adjacent flights, even performed on different days since the data acquisition from a rotor-based UAV platform could not be carried out in a single run over large areas due to restrictions in the flight range [12].

In relation to delivery times of on-demand imagery of commercial satellites and the usual times needed to implement post-fire management strategies within large burned areas [63], the length of the flight campaign (17 days) and the laboratory processing tasks (20 days) required a reasonable time. The computational demand of the project was very high due to the large amount of raw image data collected (183,500 raw images) and its ultra-high spatial resolution. The size of this dataset caused management difficulties in the laboratory in terms of data storage, backup and processing capability. This circumstance has already been reported by [20], data transfer between research teams being restricted by physical storage units or some processing options such as cloud computing. This computational demand may limit the execution of this type of projects to users who have access to high-end computers to process raw imagery. However, recent advances in computational capacity would allow a large-scale implementation of this type of workflow [64]. Other remote sensing products with reduced processing requirements such as on-demand satellite imagery offer a resolution from pan-sharpening techniques that is increasingly closer to what can be obtained with multispectral sensors on board UAVs. However, according to [65,66,67], the use of pan-sharpening techniques presents several problems such as the appearance of spatial impurities or radiometric distortions in the merged product. This type of anomaly could represent a serious problem for providing the highest radiometric and spatial accuracy for fine scale applications. On the other hand, we consider that for this type of study, a UAV is more versatile than other types of remote sensing platforms, allowing flights to be carried out in the immediate post-fire situation given the provided control of the revisit time [18]. Another possible alternative to this highly demanding processing framework could be the performance of flights in small non-adjacent surfaces within the study area to reduce the campaign effort, but it would not be feasible to obtain a multispectral product that allows extrapolation of, for example, recovery models to other areas within the study area where the flights have not been carried out. The initial georeferencing precision (RMSE_X,Y_ between 1.5–3 m and RMSE_Z_ between 2–4 m) is not an optimum result considering that some authors, such as [51], have established as low accuracy an X-Y error higher than two times the GSD and a Z error higher than three times the GSD. Single frequency GPS receivers, such as the one used in the platform, which features a light antenna and chip power limitations, typically show important drifts throughout time. This is particularly important in our case since every subproject included flights carried out at different times or even on different days due to the large size of the surveyed area. Current research on the installation of dual frequency GPS onboard UAV platforms [68] would allow for direct georeferencing the generated geomatic products without the need of GCPs [15]. The geospatial accuracy of the final georeferencing achieved by using GCPs is a good result (RMSE_X,Y_ < 30 cm and RMSE_Z_ < 55 cm) considering the great extension of the UAV survey framework and taking into account that some studies reported a decrease in accuracy with large survey areas [64]. Other studies, such as that conducted by [11], obtained similar geospatial accuracy, but in our case, the error is closer to the lower limit that approximately matches the pixel size [69]. This accuracy was highly influenced by the even distribution of the GCPs through the UAV survey framework [70,71].

Within the comparison framework of the spatial information provided by UAV and WorldView-2 imagery, the higher correlations obtained between UAV orthomosaic resampled to match WorldView-2 image resolution, confirm that in the first successional stages of the vegetation on heterogeneous burned areas, the highest spatial resolution UAV mosaic (20 cm) does not provide redundant information [12] in relation to the satellite image. In this case, the ground variability scale associated with small vegetation patches, is larger than the coarser pixel sizes. Moreover, the stronger correlation between the UAV and WorldView-2 imagery found in the visible region of the spectrum was probably due to the similar relative spectral response in that region for the two sensors [44,55]. The NDVI map comparison between the UAV and WorldView-2 imagery conducted on a heterogeneous surface within the UAV survey framework, revealed again that coarser resolution satellite imagery cannot represent the spatial variability and patterns of areas characterized by very small vegetation patches [12]. The larger variance in reflectance values for each band of the highest spatial resolution UAV orthomosaic indicates that this product may be able to capture fine-scale ground patterns because of the greater spatial information provided by the dataset, improving the interpretation of landscape features. Some authors such as [18] stated that at this spatial scale, variations in sun azimuth and elevation will create variable shadow features throughout the day. This factor may introduce reflectance variability, and therefore, distort the calculation of spectral indices in ultra- high spatial resolution images. This effect in small targets is less significant in satellite imagery given its pixel size. However, within the NDVI map comparison framework, the sun azimuth and elevation of the UAV flight approximately matches the ones in WorldView-2 capture and the variability in reflectance values of both sensors was approximately the same as for the entire study area.

## 5. Conclusions

(1) The raw imagery acquired by the Parrot SEQUOIA multispectral camera presented some undesirable anomalies such as horizontal banding noise and non-homogeneous radiometry across the image. Moreover, the irradiance sensor disconnections induced some radiometric anomalies across a small area of the multispectral mosaic that had to be discarded.
(2) The 16-bit imagery acquired on the UAV flights of the 3000 ha survey framework represents a large volume of data before processing it into a multispectral orthomosaic due to its ultra-high spatial resolution and the large size of the surveyed area. Nevertheless, this spatial resolution, which cannot be achieved with satellite platforms, could be crucial for developing spatial products to be used in post-fire management decision-making.
(3) Data processing was very labor-intensive, taking about 320 h to obtain the final multispectral orthomosaic. Due to the large imagery dataset generated on a UAV survey of a large area, the dataset processing must be subdivided regardless of the available processing capability. The obtained geospatial accuracy of the UAV multispectral orthomosaic was high (RMSE_X,Y_ < 30 cm and RMSE_Z_ < 55 cm) regarding the large extension of the surveyed area and the spatial resolution of the dataset.
(4) The spatial information provided by the ultra-high spatial resolution UAV multispectral orthomosaic was not redundant in these large and heterogeneous burned areas in comparison with high spatial resolution satellite imagery such as that provided by WorldView-2. The UAV orthomosaic could therefore improve the analysis and interpretation of fine-scale ground patterns.

## Figures and Tables

**Table 1:** Pearson correlation results between native and resampled UAV multispectral mosaics and WorldView-2 multispectral image.

	WV-2 Green	WV-2 Red	WV-2 Red Edge	WV-2 NIR1
UAV (20 cm) green	0.6832			
UAV (20 cm) red		0.7426		
UAV (20 cm) red edge			0.5873	
UAV (20 cm) NIR				0.6312
UAV (1 m) green	0.7385			
UAV (1 m) red		0.7649		
UAV (1 m) red edge			0.6134	
UAV (1 m) NIR				0.6476
UAV (2 m) green	0.7871			
UAV (2 m) red		0.7968		
UAV (2 m) red edge			0.6623	
UAV (2 m) NIR				0.6942

**Table 2:** Variance in reflectance values computed for each band of the original and resampled UAV multispectral mosaics and the WorldView-2 multispectral image.

	Green	Red	Red Edge	NIR
UAV 20 cm	0.00083	0.00170	0.00134	0.00246
UAV 1 m	0.00079	0.00161	0.00122	0.00225
UAV 2 m	0.00071	0.00146	0.00101	0.00206
WV-2	0.00068	0.00132	0.00112	0.00211

## References

[1] Seneviratne S.I., Nicholls N., Easterling D., Goodess C.M., Kanae S., Kossin J., Luo Y., Marengo J., McInnes K., Rahimi M., Field C.B., Barros V., Stocker T.F., Qin D., Dokken D.J., Ebi K.L., Mastrandrea M.D., Mach K.J., Plattner G.K., Allen S.K. (2012). Changes in climate extremes and their impacts on the natural physical environment. Managing the Risks of Extreme Events and Disasters to Advance Climate Change Adaptation.

[2] Quintano C., Fernández-Manso A., Calvo L., Marcos E., Valbuena L. (2015). Land surface temperature as potential indicator of burn severity in forest Mediterranean ecosystems. Int. J. Appl. Earth Obs..

[3] Poursanidis D., Chrysoulakis N. (2017). Remote Sensing, natural hazards and the contribution of ESA Sentinels missions. Remote Sens. Appl. Soc. Environ..

[4] Álvarez A., Gracia M., Vayreda J., Retana J. (2012). Patterns of fuel types and crown fire potential in *Pinus halepensis* forest in the Western Mediterranean Basin. For. Ecol. Manag..

[5] Vallejo R., Alloza J.A., Moreno J.M. (1998). The restoration of burned lands: The case of eastern Spain. Large Forest Fires.

[6] Tessler N., Wittenberg L., Greenbaum N. (2016). Vegetation cover and species richness after recurrent forest fires in the Eastern Mediterranean ecosystem of Mount Carmel, Israel. Sci. Total Environ..

[7] Ruíz-Gallardo J.R., Castaño S., Calera A. (2004). Application of remote sensing and GIS to locate priority intervention areas after wildland fires in Mediterranean systems: A case study from southeastern Spain. Int. J. Wildland Fire.

[8] Chu T., Guo X., Takeda K. (2016). Remote sensing approach to detect post-fire vegetation regrowth in Siberian boreal larch forest. Ecol. Indic..

[9] Viedma O., Torres I., Pérez B., Moreno J.M. (2012). Modeling plant species richness using reflectance and texture data derived from QuickBird in a recently burned area of Central Spain. Remote Sens. Environ..

[10] Jung M., Tautenhahn S., Wirth C., Kattge J. (2013). Estimating Basal Area of Spruce and Fir in Post-fire Residual Stands in Central Siberia Using Quickbird, Feature Selection, and Random Forests. Procedia Comput. Sci..

[11] Zhang J., Hu J., Lian J., Fan Z., Ouyang X., Ye W. (2016). Seeing the forest from drones: Testing the potential of lightweight drones as a tool for long-term forest monitoring. Biol. Conserv..

[12] Matese A., Toscano P., Di Gennaro S.F., Genesio L., Vaccari F.P., Primicerio J., Belli C., Zaldei A., Bianconi R., Gioli B. (2015). Intercomparison of UAV, Aircraft and Satellite Remote Sensing Platforms for Precision Viticulture. Remote Sens..

[13] Anderson K., Gaston K.J. (2013). Lightweight unmanned aerial vehicles will revolutionize spatial ecology. Front. Ecol. Environ..

[14] Tang L., Shao G. (2015). Drone Remote Sensing for Forestry Research and Practices. J. For. Res.

[15] Ribeiro-Gomes K., Hernandez-Lopez D., Ballesteros R., Moreno M.A. (2016). Approximate georeferencing and automatic blurred image detection to reduce the costs of UAV use in environmental and agricultural applications. Biosyst. Eng..

[16] Zhou J., Pavek M.J., Shelton S.C., Holden Z.J., Sankaran S. (2016). Aerial multispectral imaging for crop hail damage assessment in potato. Comput. Electron. Agric..

[17] Beaty R.M., Taylor A.H. (2001). Spatial and Temporal Variation of Fire Regimes in a Mixed Conifer Forest Landscape, Southern Cascades, California, USA. J. Biogeogr..

[18] McKenna P., Erskine P.D., Lechner A.M., Phinn S. (2017). Measuring fire severity using UAV imagery in semi-arid central Queensland, Australia. Int. J. Remote Sens..

[19] Torresan C., Berton A., Carotenuto F., Di Gennaro S.F., Gioli B., Matese A., Miglietta F., Vagnoli C., Zaldei A., Wallace L. (2016). Forestry applications of UAVs in Europe: A review. Int. J. Remote Sens..

[20] Hardin P.J., Jensen R.R. (2011). Small-Scale Unmanned Aerial Vehicles in Environmental Remote Sensing: Challenges and Opportunities. GISci. Remote Sens..

[21] Jones G.P., Pearlstine L.G., Percival H.F. (2006). An assessment of small unmanned aerial vehicles for wildlife research. Wildl. Soc. B.

[22] Koski W.R., Allen T., Ireland D., Buck G., Smith P.R., Macrander A.M., Halick M.A., Rushing C., Sliwa D.J., McDonald T.L. (2009). Evaluation of an unmanned airborne system for monitoring marine mammals. Aquat. Mamm..

[23] Israel M. A UAV-based roe deer fawn detection system. Proceedings of the International Archives of the Photogrammetry, Remote Sensing and Spatial Information Sciences, Conference on Unmanned Aerial Vehicle in Geomatics.

[24] Chabot D., Bird D.M. (2012). Evaluation of an off-the-shelf Unmanned Aircraft System for surveying flocks of geese. Waterbirds.

[25] Sarda-Palomera F., Bota G., Viñolo C., Pallarés O., Sazatornil V., Brotons L., Gomáriz S., Sarda F. (2012). Fine-scale bird monitoring from light unmanned aircraft systems. IBIS.

[26] Vermeulen C., Lejeune P., Lisein J., Sawadogo P., Bouche P. (2013). Unmanned aerial survey of elephants. PLoS ONE.

[27] Floris A., Clementel F., Colle G., Gubert F., Bertoldi L., De Lorenzi G. Estimation of Wood Volume with Photogrammetric Data Sensing from UAV on Small Surfaces: A Case Study in Trentino. Proceedings of the 16th ASITA National Conference.

[28] Getzin S., Wiegand K., Schöning I. (2012). Assessing biodiversity in forests using very high-resolution images and unmanned aerial vehicles. Methods Ecol. Evol..

[29] Wallace L., Lucieer A., Watson C., Turner D. (2012). Development of a UAV–LiDAR system with application to forest inventory. Remote Sens..

[30] Dandois J.P., Ellis E.C. (2013). High spatial resolution three-dimensional mapping of vegetation spectral dynamics using computer vision. Remote Sens. Environ..

[31] Lisein J., Pierrot-Deseilligny M., Bonnet S., Lejeune P. (2013). A Photogrammetric Workflow for the Creation of a Forest Canopy Height Model from Small Unmanned Aerial System Imagery. Forests.

[32] Puliti S., Orka H.O., Gobakken T., Naesset E. (2015). Inventory of small forest areas using an Unmanned Aerial System. Remote Sens..

[33] Fritz A., Kattenborn T., Koch B. UAV-based photogrammetric point clouds-tree stem mapping in open stands in comparison to terrestrial laser scanner point clouds. Proceedings of the UAV-g2013.

[34] Gini R., Passoni D., Pinto L., Sona G. (2014). Use of Unmanned Aerial Systems for Multispectral Survey and Tree Classification: A Test in a Park Area of Northern Italy. Eur. J. Remote Sens..

[35] Jaakkola A. (2015). Low-cost Mobile Laser Scanning and its Feasibility for Environmental Mapping. Ph.D. Dissertation.

[36] Michez A., Piégay H., Lisein J., Claessens H., Lejeune P. (2016). Classification of Riparian Forest Species and Health Condition Using Multi-Temporal and Hyperspatial Imagery from Unmanned Aerial System. Environ. Monit. Assess..

[37] Aicardi I., Garbarino M., Lingua A., Lingua E., Marzano R., Piras M. Monitoring Post-Fire Forest Recovery Using Multitemporal Digital Surface Models Generated from Different Platforms. Proceedings of the EARSeL Symposium.

[38] Fraser R.H., van der Sluijs J., Hall R.J. (2017). Calibrating Satellite-Based Indices of Burn Severity from UAV-Derived Metrics of a Burned Boreal Forest in NWT, Canada. Remote Sens..

[39] Cruz H., Eckert M., Meneses J., Martínez J.F. (2016). Efficient Forest Fire Detection Index for Application in Unmanned Aerial Systems (UASs). Sensors.

[40] Pérez-Ortiz M., Peña J.M., Gutiérrez P.A., Torres-Sánchez J., Hervás-Martínez C., López-Granados F. (2016). Selecting patterns and features for between- and within- crop-row weed mapping using UAV-imagery. Expert Syst. Appl..

[41] Misopolinos L., Zalidis C.H., Liakopoulos V., Stavridou D., Katsigiannis P., Alexandridis T.K., Zalidis G. Development of a UAV system for VNIR-TIR acquisitions in precision agriculture. Proceedings of the Third International Conference on Remote Sensing and Geoinformation of the Environment.

[42] Tian J., Wang L., Li X., Gong H., Shi C., Zhong R., Liu X. (2017). Comparison of UAV and WorldView-2 imagery for mapping leaf area index of mangrove forest. Int. J. Appl. Earth Obs..

[43] Calvo L., Santalla S., Valbuena L., Marcos E., Tárrega R., Luis-Calabuig E. (2008). Post-fire natural regeneration of a *Pinus pinaster* forest in NW Spain. Plant Ecol..

[44] Parrot. https://community.parrot.com/t5/Sequoia/bd-p/Sequoia.

[45] Santesteban L.G., Di Gennaro S.F., Herrero-Langreo A., Miranda C., Royo J.B., Matese A. (2017). High-resolution UAV-based thermal imaging to estimate the instantaneous and seasonal variability of plant water status within a vineyard. Agric. Water Manag..

[46] Kelcey J., Lucieer A. (2012). Sensor Correction of a 6-Band Multispectral Imaging Sensor for UAV Remote Sensing. Remote Sens..

[47] Burkart A., Aasen H., Alonso L., Menz G., Bareth G., Rascher U. (2015). Angular Dependency of Hyperspectral Measurements over Wheat Characterized by a Novel UAV Based Goniometer. Remote Sens..

[48] Koik B.T., Ibrahim H. A literature survey on blur detection algorithms for digital imaging. Proceedings of the 1st International Conference on Artificial Intelligence, Modelling and Simulation.

[49] Pix4D. https://pix4d.com/product/pix4dmapper-photogrammetry-software/.

[50] McGlone J.C. (2013). Manual of Photogrammetry.

[51] Ruzgiené B., Berteška T., Gečyte S., Jakubauskienė E., Aksamitauskas V.C. (2015). The surface modelling based on UAV Photogrammetry and qualitative estimation. Measurement.

[52] Zahawi R.A., Dandois J.P., Holl K.D., Nadwodny D., Reid J.L., Ellis E.C. (2015). Using lightweight unmanned aerial vehicles to monitor tropical forest recovery. Biol. Conserv..

[53] PNOA. http://pnoa.ign.es/caracteristicas-tecnicas.

[54] ESRI. http://desktop.arcgis.com/es/arcmap/10.3/main/get-started/whats-new-in-arcgis-1031.htm.

[55] DigitalGlobe. http://global.digitalglobe.com.

[56] Matthew M., Adler-Golden S., Berk A., Felde G., Anderson G., Gorodetzky D., Paswaters S., Shippert M. Atmospheric correction of spectral imagery: Evaluation of the FLAASH algorithm with AVIRIS data. Proceedings of the 32nd Applied Imagery Pattern Recognition Workshop.

[57] ENVI. http://www.harrisgeospatial.com/SoftwareTechnology/ENVI.aspx.

[58] Laliberte A.S., Goforth M.A., Steele C.M., Rango A. (2011). Multispectral Remote Sensing from Unmanned Aircraft: Image Processing Workflows and Applications for Rangeland Environments. Remote Sens..

[59] Ortega-Terol D., Hernandez-Lopez D., Ballesteros R., Gonzalez-Aguilera D. (2017). Automatic Hotspot and Sun Glint Detection in UAV Multispectral Images. Sensors.

[60] Tellidis I., Levin E. Photogrammetric Image Acquisition with Small Unmanned Aerial Systems. Proceedings of the ASPRS 2014 Annual Conference Proceedings.

[61] Stark B., Zhao T., Chen Y. An analysis of the effect of the bidirectional reflectance distribution function on remote sensing imagery accuracy from Small Unmanned Aircraft Systems. Proceedings of the International Conference on Unmanned Aircraft Systems (ICUAS).

[62] Roy D.P., Li J., Zhang H.K., Yan L., Huang H., Li Z. (2017). Examination of Sentinel-2A multi-spectral instrument (MSI) reflectance anisotropy and the suitability of a general method to normalize MSI reflectance to nadir BRDF adjusted reflectance. Remote Sens. Environ..

[63] Taboada A., Tárrega R., Marcos E., Valbuena L., Suárez-Seoane S., Calvo L. (2017). Fire recurrence and emergency post-fire management influence seedling recruitment and growth by altering plant interactions in fire-prone ecosystems. For. Ecol. Manag..

[64] Koci J., Jarihani B., Leon J.X., Sidle R.C., Wilkinson S.N., Bartley R. (2017). Assessment of UAV and Ground-Based Structure from Motion with Multi-View Stereo Photogrammetry in a Gullied Savanna Catchment. ISPRS Int. Geo-Inf..

[65] Laporterie-Dejean F., Boissezon H., Flouzat G., Lefevre-Fonollosa M.J. (2005). Thematic and statistical evaluations of five panchromatic/multispectral fusion methods on simulated PLEIADES-HR images. Inf. Fusion.

[66] Thomas C., Ranchin T., Wald L., Chanussot J. (2008). Synthesis of multispectral images to high spatial resolution: A critical review of fusion methods based on remote sensing physics. IEEE Trans. Geosci. Remote Sens..

[67] Tu T.M., Su S.C., Shyu H.C., Huang P.S. (2001). A new look at IHS-like image fusion methods. Inf. Fusion.

[68] Karl-Lehmann J.R., Nieberding F., Prinz T., Knoth C. (2015). Analysis of Unmanned Aerial System-Based CIR Images in Forestry—A New Perspective to Monitor Pest Infestation Levels. Forests.

[69] Mesas-Carrascosa F.J., Torres-Sánchez J., Clavero-Rumbao I., García-Ferrer A., Peña J.M., Borra-Serrano I., López-Granados F. (2015). Assessing Optimal Flight Parameters for Generating Accurate Multispectral Orthomosaicks by UAV to Support Site-Specific Crop Management. Remote Sens..

[70] Shahbazi M., Sohn G., Théau J., Menard P. (2015). Development and Evaluation of a UAV-Photogrammetry System for Precise 3D Environmental Modeling. Sensors.

[71] Harwin S., Lucieer A. (2012). Assessing the Accuracy of Georeferenced Point Clouds Produced via Multi-View Stereopsis from Unmanned Aerial Vehicle (UAV) Imagery. Remote Sens..

